# The Advertising Doctor

**Published:** 1901-11

**Authors:** 


### THE ADVERTISING DOCTOR.

In reply to a virulent editorial attack upon "medical ethics, contained in the *Chicago Tribune, American Medicine* says: "The *Tribune's* mistake is based upon the stupidity of supposing we have anything that can be sold, such as secret prescriptions, nostrums, discoveries and lies—things which the *Tribune* evidently likes. The physician has nothing of the kind for sale. He has only his medical, diagnostic and surgical skill to offer to the sick and their friends. In other words, he has only his character and ability to give. Does any man who has character and ability go around advertising the fact? If he does he is at once looked upon as a blatant egotist. If he possesses the superiority, he is the last man to brag about it. Do judges, lawyers, ministers, railway presidents and managers advertise in the *Tribune* their peculiar excellencies? We have never seen the advertisements of even poor reporters and yellow journal editors. Would not the advertisements for "positions," and the parading of their abilities by themselves furnish at once indisputable proof that they were wanting precisely in those very qualities of which they boasted? Self-laudation should be a trick which even the *Chicago Tribune* should be capable of exposing instead of praising."

This states the case admirably. It is to be regretted that such articles do not find their way into the lay press, for the medical profession cannot hope to wage successful war against the advertising quack until the public is made to understand why the charlatan advertises and why the honest physician does not. The atmosphere of mystery with which Christian scientists, magnetic healers, and the like, surround themselves, affords sufficient protection from the criticism which would be likely to have weight with their feeble minded followers. But not so secure is the self-styled eminent universal specialist, who depends for his success upon lying, and often obscene, advertisements, the absurdity of which can be demonstrated in a way that will convince even the densest intellect. With him the sun of prosperity will have forever set when it becomes a matter of common knowledge that his object in publishing lists of symptoms (which he knows the lay mind cannot properly interpret) is to mislead the imaginative and credulous into believing themselves to be the victims of disease, and that in publishing his absurd claims to superior skill and knowledge he deliberately sacrifices both honor and self-respect for cash. Even now the majority of intelligent laymen understand that the doctor who advertises in the usual way is at once a disgusting egotist and a designing scoundrel, whose privilege to impose upon the ignorant members of society should be taken from him by due process of law. R.

